# Doing simple things well can achieve significant benefits

**DOI:** 10.1016/j.clinme.2024.100219

**Published:** 2024-06-25

**Authors:** Ponnusamy Saravanan

**Affiliations:** a Warwick Applied Health, Warwick Medical School, University of Warwick, Coventry, United Kingdom; b Diabetes, Endocrinology and Metabolism, George Eliot Hospital NHS Trust, Nuneaton, United Kingdom

Medicine is a profession that not only provides great job satisfaction, but also continually challenges healthcare professionals to learn daily. This ongoing learning facilitates constant improvements in our evidence-based approach to patient care. This issue of *Clinical Medicine* includes several interesting case reports,^1^, ^2^, ^3^, ^4^, ^5^ original articles on strategies for improving ethnic representation in neurological studies,^6^ a global comparison of early warning scores in prehospital trauma,^7^ the importance of repeated clinical examinations in managing fever of unknown origin,^8^ the use of outpatient parenteral antibiotic treatment for infective endocarditis,^9^ and a consensus report on how to measure quality of care in ‘same-day emergency care’ units across the UK.^10^

Digital health and data science are here to stay. This issue features an interesting study that uses these methodologies to enhance medical undergraduate education. Notably, this research was co-developed with medical students, which is likely to foster higher engagement and potentially better learning outcomes.^11^ Additionally, the issue includes three opinion/foundation column articles: the current status of obesity management and the need to revisit current National Institute for Health and Care Excellence (NICE) guidelines,^12^ the experiences of a foundation doctor on an aeroplane^13^ and the significance of correctly interpreting the CMO's report on the increasing number of older people in rural areas.^14^

While I believe that every article in this issue contributes valuable insights across various medical disciplines, my top choice highlights how implementing simple, well-known practices can yield significant benefits. The systematic review by Awad *et al* ^15^ demonstrates that intravenous iron not only significantly improves quality of life, but also positively impacts key outcomes including the 6-minute walk test, left ventricular ejection fraction, heart failure-related hospitalisations and all-cause mortality. Heart failure with reduced ejection fraction can be devastating for individuals, often resulting in repeated hospitalisations and a 5-year mortality worse than many cancers. More than 50% of patients with heart failure have iron deficiency anaemia, which is often treated with oral therapy, and these patients could benefit from this approach. The authors call for comparative studies using different iron formulations and emphasise the importance of establishing parenteral iron therapy as the standard of care for all heart failure patients with iron deficiency. Consistently doing simple things right can indeed lead to meaningful improvements.
